# Antibody-Drug Conjugates (ADC) Against Cancer Stem-Like Cells (CSC)—Is There Still Room for Optimism?

**DOI:** 10.3389/fonc.2019.00167

**Published:** 2019-03-29

**Authors:** Fabrizio Marcucci, Carmelo Antonio Caserta, Elisabetta Romeo, Cristiano Rumio

**Affiliations:** ^1^Department of Pharmacological and Biomolecular Sciences, University of Milan, Milan, Italy; ^2^Fondazione per la Medicina Solidale, Reggio Calabria, Italy

**Keywords:** cancer stem cell, epithelial-mesenchymal transition, resistance, antibody-drug conjugate, resting, proliferating

## Abstract

Cancer stem-like cells (CSC) represent a subpopulation of tumor cells with peculiar functionalities that distinguish them from the bulk of tumor cells, most notably their tumor-initiating potential and drug resistance. Given these properties, it appears logical that CSCs have become an important target for many pharma companies. Antibody-drug conjugates (ADC) have emerged over the last decade as one of the most promising new tools for the selective ablation of tumor cells. Three ADCs have already received regulatory approval and many others are in different phases of clinical development. Not surprisingly, also a considerable number of anti-CSC ADCs have been described in the literature and some of these have entered clinical development. Several of these ADCs, however, have yielded disappointing results in clinical studies. This is similar to the results obtained with other anti-CSC drug candidates, including native antibodies, that have been investigated in the clinic. In this article we review the anti-CSC ADCs that have been described in the literature and, in the following, we discuss reasons that may underlie the failures in clinical trials that have been observed. Possible reasons relate to the biology of CSCs themselves, including their heterogeneity, the lack of strictly CSC-specific markers, and the capacity to interconvert between CSCs and non-CSCs; second, inherent limitations of some classes of cytotoxins that have been used for the construction of ADCs; third, the inadequacy of animal models in predicting efficacy in humans. We conclude suggesting some possibilities to address these limitations.

## Cancer Stem-Like Cells (CSC), a Tumor Cell Subpopulation With Peculiar Properties

CSCs are carcinoma cells that self-renew and give rise to differentiated tumor cells. CSCs by themselves, however, can arise from differentiated tumor cells when these cells undergo an epithelial-mesenchymal transition (EMT) (1). EMT involves changes that lead to loss of cell-cell adhesion and cell polarity, with acquisition of migratory and invasive properties (2). EMT encompasses a continuum of states from a fully epithelial to a fully mesenchymal phenotype, passing through intermediate, hybrid states (3). Interestingly, it has recently been shown that acquisition of tumor-initiating potential is one of the earliest functions gained during EMT, while other functions, like invasiveness and metastatic potential are acquired during later stages (4). These results reinforce the close relationship between EMT and CSCs (1).

In addition to their tumor-initiating potential, CSCs possess also other functions that they share with EMT tumor cells, most notably drug resistance (5). Drug resistance implies that tumor cells survive drug treatment and become enriched in the tumor cell population. In fact, one key assay to ascertain the *in vivo* efficacy of anti-CSC compounds is to test the number of tumor cells that are required in order to initiate tumor growth in animal models before and after drug treatment (6).

Considerable efforts have been devoted to the phenotypic characterization of CSCs, in particular the identification of markers that distinguish CSCs from normal stem cells and the bulk of differentiated tumor cells. Overall, it has been difficult to define CSCs on the basis of their phenotypic profile (5). Thus, a large number of cell surface molecules that are expressed on CSCs have been identified; CD44, CD47, CD33, CD133, CXC chemokine receptor (CXCR) 4, and CD26 are some of these markers. Most of them, however, are not CSC-specific and in some cases are even ubiquitously expressed (e.g., CD44, CD47) (7). Some markers have a more restricted expression and/or are overexpressed on CSCs; these have been used as targets for ADCs, as will be discussed in the following.

The plasticity of CSCs is reflected also by the large number of signaling pathways that are involved in the induction and maintenance of CSCs. Given the functional relationship between CSCs and normal stem cells, the role of signaling pathways involved in the physiology of normal stem cells, such as WNT, Notch, and Hedgehog (Hh), has been investigated with particular attention (8).

Eventually, also post-transcriptional regulation contributes to the homeostasis and functions of CSCs. These include RNA modifications, RNA-binding proteins, mircoRNAs and long non-coding RNAs (9).

As regards the generation of CSCs from differentiated tumor cells, similarly to cells that undergo an EMT, tumor-initiating potential can be acquired when one of three different events occur. First, in response to stressors from the tumor microenvironment like hypoxia, low pH, immune responses, mechanical stress, and antitumor drugs (10, 11). Second, stressor-promoted epigenetic changes that induce heritable effects allowing retention of the mesenchymal state even when the stressors are no longer present (12, 13). Third, stimulus-independent activation of signaling pathways, owing to activating mutations or overexpression of pathway components (14, 15). Intuitively, these events are not mutually exclusive and may differ quantitatively and qualitatively in different tumors and, over time, even within the same tumor. Moreover, some of these events (e.g., stressor-induced responses) can be reversible and, consequently, CSCs can revert back to a differentiated phenotype, as already referred to above. Vice versa, tumor cells that have regained an epithelial and a non-CSC phenotype can undergo a *de novo* switch toward a more mesenchymal tumor-initiating phenotype, even after drug-induced depletion of CSCs. As such, depletion of CSCs is by no means a conclusive effect but, rather, a transient elimination of tumor cells engaged in the replenishment of a tumor cell population of epithelial phenotype.

## Antibody-Drug Conjugates (ADC), Tools for the Selective Elimination of Tumor Cells

**Figure 1:**
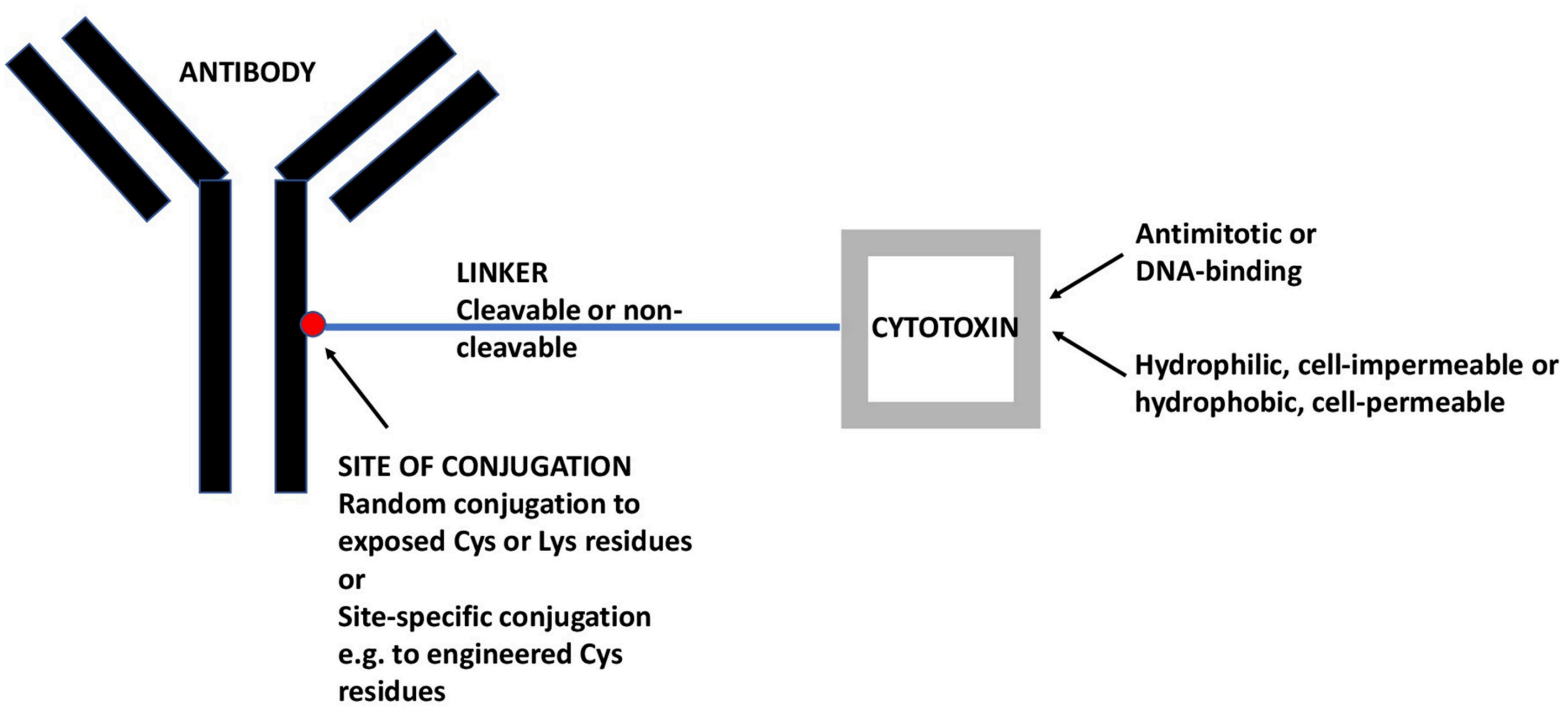
A Schematic View of ADCs and its Individual Components. The mAb targets a tumor-associated antigen, in the present case an antigen that is preferentially expressed on CSCs. The linker may be cleavable (e.g., acid-sensitive or dipeptide) or non-cleavable (e.g., maleimidocaproyl). The cytotoxin may be an antimitotic drug, active only on proliferating cells or a DNA-binding drug, active also on quiescent cells. Moreover, the cytotoxin may be hydrophilic and act only within the internalizing cell or it may be hydrophobic and act also on nearby cells, whether antigen-positive or –negative (so-called bystander effect). ADC, antibody-drug conjugate; CSC, cancer stem-like cell; mAb, monoclonal antibody.

ADCs comprise a monoclonal antibody (mAb) against a tumor-associated antigen, a covalent linker, and a cytotoxic payload (16). Figure 1 gives a schematic view of an ADC and its individual components as will be discussed in the following. In most cases, ADCs are internalized upon binding to the cognate antigen and the cytotoxic payload is released, causing cell death. The targeted delivery of cytotoxins to tumor cells allows for the maximum efficacy and minimal toxicity.

The mAb should recognize an antigen expressed on the largest possible fraction of tumor cells and the smallest possible fraction of normal cells (17). With the exception of hematological malignancies, there is no known antigen that is homogeneously expressed on all tumor cells because the tumor cell population is, in itself, heterogeneous and composed, to varying degrees, of antigen-positive or strongly positive (preferably the vast majority) and antigen-negative or weakly positive tumor cells. Incidentally, the existence of phenotypic tumor cell heterogeneity justifies the aim of generating anti-CSC ADCs, because this rests on the assumption that CSCs display an antigenic profile that differs qualitatively and/or quantitatively from that of non-CSCs and, thus, may escape cytotoxicity induced by ADCs that target non-CSCs. MAbs that are currently used for the engineering of ADCs are of human origin or are humanized murine antibodies (18, 19) in order to minimize antigenicity and the induction of anti-drug antibodies.

The second component, the linker, is important for the stability of the ADC. It should be sufficiently stable to negate systemic release of the cytotoxic payload, but sufficiently labile to allow intracellular release, in most cases within the lysosomal compartment. Dipeptide linkers like valine-citrulline are typical examples of linkers that are cleaved with good selectivity within the lysosomal compartment (20). However, for some ADCs also non-cleavable linkers are used and, in this case, the cytotoxic payload is released as an amino acid conjugate upon degradation of the antibody. These linkers can be used if the drug-linker-amino acid residue conjugate retains drug activity (21). Ado-trastuzumab emtansine is an example of an ADC with a non-cleavable linker yielding a lysine-linker-cytotoxic (DM1) complex (22).

Cytotoxins used for ADC synthesis are highly potent because of the limited number of payloads that each individual antibody molecule can carry. Most ADC payloads belong to two mechanistic classes. The first are antimitotic, tubulin-binding cytotoxins like auristatins and maytansines. The second are DNA-binding, cell cycle-independent cytotoxins like calicheamicins and pyrrolobenzodiazepines (PBD).

Once released, a cytotoxin may be cell-impermeable [e.g., more hydrophilic cytotoxins like monomethyl auristatin (MMA) F] and exert its cytotoxic effect exclusively on a single cell, or it may penetrate the cell membrane and exert bystander killing also on nearby cells (e.g., more lipophilic cytotoxins like MMAE) (23), whether or not these cells are positive for the target antigen. This potential advantage of cell-permeable cytotoxins must be weighed against the possibility of enhanced systemic toxicity due to back-flow of the liberated cytotoxin into the systemic circulation.

Until recently, most ADCs were generated by random conjugation of the linker to available cysteine or lysine residues on the antibody. This approach leads to ADC mixtures with different drug-antibody ratios (DAR) with the individual components having distinct properties that may lead to suboptimal *in vivo* efficacy of the final mixture. In recent years, site-specific conjugation methods have been developed, which yield ADCs with defined DARs. These ADCs have been shown to possess larger therapeutic windows and to be better tolerated than randomly-conjugated ADCs (24, 25).

An important question to answer in the present context is as to why an ADC should be preferred over a native mAb as an anti-CSC agent. The question is not trivial since also native antibodies are endowed with cytotoxic potential. In fact, mechanisms like antibody-dependent cellular cytotoxicity, complement-dependent cytotoxicity or antibody-dependent cellular phagocytosis contribute, to varying degrees, to the efficacy of an antitumor mAb (26). The answer lies in the greater antitumor efficacy that ADCs have demonstrated *in vivo* compared to native, equivalent mAbs (27) and the efficacy that ADCs, like ado-trastuzumab emtansine (T-DM1), have shown in tumors that were *ab initio* unresponsive or had become unresponsive to the native equivalent (trastuzumab) (28). It is to note, however, that the cytotoxic potential of the native mAb has been shown to contribute to the overall efficacy of ADCs (29).

## Anti-CSC ADCs That Have Been Described in the Literature

In the following we will discuss individual anti-CSC ADCs that have been reported in the scientific literature. In some instances, more than one ADC has been described against an individual target and we will briefly address the properties of each of these conjugates. We will first discuss those ADCs that target markers expressed on CSCs. In a following section we will discuss antitumor ADCs that were shown to have anti-CSC activity at a later stage.

### ADCs Against Markers Expressed on CSC

#### Anti-delta-like Ligand (DLL) 3 ADC

This target is of particular interest in these days, because the development of an ADC against DLL3, rovalpituzumab tesirine, which had reached phase III clinical studies, has failed to show benefit in third-line small-cell lung cancer (SCLC) (30).

Rovalpituzumab tesirine had been constructed for the targeting of CSCs of high-grade pulmonary endocrine tumors (31). These tumors include SCLC and large cell neuroendocrine carcinoma, highly malignant neoplasms with few and inefficacious therapeutic options (32). CSCs of SCLC are thought to arise from normal pulmonary neuroendocrine cells, the portion of the diffuse neuroendocrine system found in the respiratory epithelium.

DLL3 is a ligand of the mammalian Notch family that localizes to the Golgi apparatus without being able to activate Notch signaling like other Notch ligands. Rather, DLL3 appears to inhibit Notch pathway activation by interacting with Notch and the Notch ligand DLL1, thereby preventing their localization to the cell surface (33). Notch activation in neuroendocrine tumors has been shown to suppress tumor growth (34). Expression data showed that DLL3 mRNA is overexpressed in primary SCLC tumors, SCLC patient-derived xenografts (PDX), conventional SCLC cell lines, and large-cell neuroendocrine carcinoma PDXs, whereas mRNA expression in normal tissues appears limited primarily to the brain (31). Moreover, in contrast to normal cells, DLL3 was detectable at the surface of neuroendocrine tumor cells.

On the basis of these observations, DLL3 was selected as a target for the construction of an ADC. The ADC, SC16LD6.5 (later rovalpituzumab tesirine) is composed of a humanized anti-DLL3 mAb, conjugated to a PBD dimer. The payload was conjugated to cysteine residues on the mAb via a valine-alanine dipeptide, with a mean DAR of 2. SC16LD6.5 induced durable tumor regression in multiple PDX models after a single course of therapy and in a manner independent of their sensitivity to standard-of-care (SOC) chemotherapy. Lack of tumor recurrence was shown being due to depletion of DLL3^+^ CSCs. In contrast, SOC chemotherapy did not reduce the frequency of CSCs nor provided durable responses in spite of efficacious tumor cell debulking. DLL3, however, was not CSC-specific since its expression was seen throughout the tumor, with most cells expressing it to some degree. For this reason, the rapid tumor debulking seen with SC16LD6.5 was likely due to DLL3 expression on most tumor cells. Eventually, SC16LD6.5 was efficacious also in a chemorefractory tumor model suggesting that patients with tumors resistant to SOC will be responsive to this conjugate. Of note, administration of the unconjugated anti-DLL3 mAb, even at high doses, or the equivalent dose of free PBD dimer had little or no effect on tumor growth, thereby supporting the superiority of the antitumor effects of the ADC.

Rovalpituzumab tesirine has entered numerous clinical studies and has progressed until phase III in patients with advanced SCLC after disease progression following SOC chemotherapy protocols (Table 1). Other clinical trials are earlier stage in SCLC alone (NCT02674568, NCT02819999, NCT03319940, NCT02874664) or in SCLC and other advanced solid tumors expressing cell surface DLL3 (NCT02709889). As referred to in the beginning of this section, rovalpituzumab tesirine failed to show benefit in one of the SCLC studies.

**Table 1:** Anti-CSC ADCs.

**Target: ADC name**	**Linker: Cleavable/uncleavable**	**Cytotoxin: Antimitotic, DNA binder**	**Developmental stage Stage, ClinicalTrials.gov identifiers, results if available**	**References**
**ADCs AGAINST MARKERS EXPRESSED ON CSC**				
DLL3: rovalpituzumab tesirine	Cleavable (dipeptide)	DNA binder (PBD dimer)	Phase III, NCT03061812, vs. topotecan in DLL3^+^ advanced or metastatic SCLC at first disease progression after platinum chemotherapy Phase III, NCT03334487, evaluating the safety for third-line and later treatment of relapsed or refractory SCLC Phase II, NCT02674568, as third-line and later treatment for subjects with relapsed or refractory DLL3^+^ SCLC Other phase I/II studies in patients with DLL3^+^ SCLC (NCT02819999, NCT02874664) or SCLC and other solid tumors (NCT02709889)	(30, 31)
Protein tyrosine kinase 7 (PTK7): PF-06647020	Cleavable (dipeptide)	Antimitotic (Aur0101)	Safety study, NCT03243331: with gedatolisib in TNBC Phase I, NCT02222922: in adult patients with advanced solid tumors	(35)
Ephrin-A4 (EFNA4): PF-06647263	Cleavable (hydrazone)	DNA binder (calicheamicin)	Phase I, NCT02078752: in patients with advanced solid tumors	(36)
IL-3 receptor α chain (CD123): SGN-123	Cleavable (dipeptide)	DNA binder (PBD dimer)	Phase I, NCT02848248, in AML patients. Study terminated, presumably no longer in active development.	(37)
5T4: PF-06263507	Non-cleavable (maleimidocaproyl)	Antimitotic (MMAF)	Phase I, NCT01891669, no objective responses were observed	(38, 39)
5T4: MEDI-0641	Cleavable (dipeptide)	DNA binder (PBD dimer) Antimitotic (tubulysin)	Not reported	(40, 41)
5T4: H6-DM4	Cleavable (SPDB)	Antimitotic (DM4)	Not reported	(42)
LGR5	Cleavable (dipeptide) Non-cleavable (malemidopropionyl)	Antimitotic (MMAE) Antimitotic (MMAE)	Not reported Not reported	(43)
LGR5	Cleavable (dipeptide) Cleavable (acid-sensitive)	Antimitotic (MMAE) DNA binder (PNU159682)	Not reported Not reported	(44)
**ANTITUMOR ADCs THAT WERE SHOWN TO HAVE ANTI-CSC ACTIVITY AT A LATER STAGE**				
HER2: T-DM1, ado-trastuzumab emtansine	Non-cleavable	Anti-mitotic (DM1)	FDA-approved for the treatment of HER2-positive metastatic breast cancer.	(45)
CD33: gemtuzumab ozogamicin	Cleavable (hydrazone)	DNA binder (calicheamicin)	FDA approval in 2000 for the treatment of AML. Voluntarily withdrawn in 2010 due to safety concerns. Recently reapproved.	(46)
NCAM (CD56): lovortuzumab mertansine	Cleavable	Anti-mitotic (DM1)	It was in development as antitumor agent, not specifically as anti-CSC agent. Development now halted due to disappointing results in lung cancer patients	(47, 48)

*ADC, antibody-drug conjugate; AML, acute myeloid leukemia; CSC, cancer stem-like cells; DLL3, Delta-like ligand 3; EFNA4, Ephrin-A4; FDA, Food and Drug administration; HER2, human epidermal growth factor receptor 2; LGR5, leucine-rich repeat-containing, G protein-coupled receptor 5; MMAE, monomethyl auristatin E; NCAM, neural cell-adhesion molecule; PBD, pyrrolobenzodiazepine; PTK7, protein tyrosine kinase 7; SCLC, small-cell lung cancer*.

#### Anti-protein Tyrosine Kinase 7 (PTK7) ADC

PTK7 has been identified as a CSC marker and potential target for ADCs (35). It is a conserved member of the pseudokinase family of receptor protein tyrosine kinases. The lack of kinase activity is the consequence of substitutions at residues in the kinase domain. Oncogenic functions of PTK7, including resistance to chemotherapy, have been reported for various carcinomas and acute myeloid leukemia (AML) (49). PTK7 was shown to be overexpressed in tumors vs. normal tissues, and enriched in CSCs of different tumor types. PTK7 expression within each tumor is heterogeneous, and the extent of heterogeneity varies from tumor to tumor. PTK7 staining was also observed in some normal tissues, including esophagus, urinary bladder, kidney, mammary gland, lung, ovary, uterus, and digestive tract (35). The expression profile of PTK7 prompted the generation an anti-PTK7 ADC (35). The choice of an ADC was also dictated by the lack of catalytic function of PTK7, making it an unsuitable target for inhibitor antibodies or small molecules.

The anti-PTK7 ADC that was constructed, PF-06647020, comprises a humanized anti-PTK7 mAb, a cleavable dipeptide (valine-citrulline) linker, and Aur0101, an auristatin microtubule inhibitor. PF-06647020 induced sustained regressions in PDX models and reduced the frequency of CSCs. In addition to CSC depletion, PF-06647020 may also have additional antitumor mechanisms of action, including angiogenesis inhibition and stimulation of immune cells. These activities may be facilitated by the bystander effect of the membrane-permeable hydrophobic auristatin payload. Importantly, despite the expression of PTK7 in certain normal tissues, no target-dependent toxicities were observed in monkeys, possibly because microtubule inhibitors require high antigen expression and actively cycling cells to exert a cytotoxic effect (50).

Two clinical studies with PTK7-ADC/PF-06647020 are currently ongoing: a safety study of the combination gedatolisib plus PF-06647020 for metastatic triple-negative breast cancer (TNBC) (NCT03243331), and a phase I study of PF-06647020 in adult patients with advanced solid tumors (NCT02222922).

#### Anti-Eph-A4 (EFNA4) ADC

Ephrin receptors (Eph) are the largest family of receptor tyrosine kinases in the human genome and, together with their ligands, have been implicated in the development of breast cancer. Consequently, numerous therapeutics, mostly tyrosine kinase inhibitors are being actively developed to inhibit the function of this ligand/receptor family. A significant limitation of currently developed drug candidates is represented by the vast functional redundancy of this family, while general inhibition of ephrin receptors is toxic (51). In contrast, ADCs prescind from the functional role of ligand/receptor pairs and exert cytotoxic effects only on those target-expressing cells that internalize them.

MRNA expression of one of these receptors, *EFNA4*, was found to be elevated in CSCs compared to non-tumorigenic cells and normal tissues. Protein analysis of normal organs, primary breast tumor specimens and TNBC PDX tumor models demonstrated that EFNA4 was elevated in TNBC vs. normal tissues and other subtypes of breast cancer (36). This expression pattern suggested the possibility of targeting EFNA4 with an ADC.

In order to target EFNA4, the ADC PF-06647263 was constructed. It is composed of an anti-EFNA4-specific, humanized mAb, a hydrazone linker and a calicheamicin payload. Conjugation was to lysine residues of the antibody and yielded an average DAR of 4.6. Calicheamicin was selected as payload because of the presence, within the CSC population, of quiescent as well as cycling cells (67). Quiescent cells are resistant to antimitotic, cell-cycle-dependent microtubule inhibitors like auristatins and maytansines. Calicheamicins, on the other hand, are DNA-binding drugs that are cytotoxic independently of the cell cycle status.

PF-06647263 induced significant tumor regression in TNBC xenografts. The most robust responses were observed in non-claudin low TNBCs, with complete responses observed in several cases. This result correlates with the increased EFNA4 expression observed in this breast cancer subtype. EFNA4 expression was also elevated on a subset of ovarian cancers and PF-06647263 induced sustained regression also on xenografts of these tumors.

A phase I study has been performed with PF-06647263 in patients with advanced solid tumors (NCT02078752). The study has been completed and no other studies are currently ongoing.

Of note, also another anti-Eph ADC has been reported in the literature (MEDI-547) (52). This ADC targets EFNA A2 and showed substantial activity in *in vivo* models of endometrial carcinoma. However, no evidence was brought that this ADC has some preferential activity on CSCs. A clinical study with this ADC has evidenced early and serious adverse events and has led to the discontinuation of its development (53).

#### An Anti-CD123 ADC

CD123 is the α chain of the interleukin (IL)-3 receptor. Upon binding of IL-3, CD123 heterodimerizes with the β subunit of the IL-3 receptor and gives rise to intracellular signals promoting cell survival and proliferation (54). CD123 is expressed on the surface of myeloblasts and leukemia stem cells (LSC, the equivalent of CSCs for hematologic malignancies) of AML patients (55, 56). These cells have been associated with chemotherapy resistance, persistence of minimal residue disease and unfavorable prognosis (57, 58). CD123 is expressed at very low levels or is absent from normal hematopoietic stem cells, thereby offering the possibility of targeting AML stem cells while sparing normal stem cells (59).

The generation and preclinical investigation of an anti-CD123 ADC has recently been reported (37). This ADC, dubbed SGN-CD123A, is composed of a humanized anti-CD123 mAb with engineered cysteines for site-specific conjugation, a valine-alanine dipeptide linker and a PBD dimer payload. *In vitro*, SGN-CD123A had potent cytotoxic effects on most CD123^+^ AML cells lines and primary samples from AML patients. SGN-CD123A was highly active in various leukemia models and led to eradication in a disseminated AML model. SGN-CD123A was also tested in combination with the fms-like tyrosine kinase 3 (FLT3) inhibitor quizartinib. FLT3 inhibitors are in development for FLT3-mutated AML patients, but responding patients invariably develop resistance. SGN-CD123A enhanced the activity of quizartinib in FLT3-mutated xenograft models.

A phase I clinical trial has been performed with SGN-CD123A in AML patients (NCT02848248). This study, however, has been terminated, and on the company's website this product is not mentioned.

#### Anti-5T4 ADCs

5T4, or trophoblast glycoprotein, is a 72-kDa, N-glycosylated transmembrane protein. The extracellular domain contains leucine-rich repeats, which are commonly associated with protein-protein interactions. 5T4 is expressed in normal progenitor cells during embryonic development where it functions in EMT and cell migration (60). It is an oncofetal antigen with high expression in many types of carcinomas and low expression in normal tissues. 5T4 has been found to be overexpressed in CSCs in non-small cell lung cancer (NSCLC) and other cancers (61) compared to differentiated tumor cells. 5T4-overexpressing cells also show increased expression of EMT markers and have increased tumor-initiating potential (61). Moreover, as expected for EMT tumor cells and CSCs, 5T4 overexpression is associated with advanced-stage disease, drug resistance and worse prognosis in several solid tumors (62–64).

Several ADCs targeting 5T4 have been described in the literature. The first, PF-06263507, comprises a humanized anti-5T4 mAb linked to the tubulin inhibitor MMAF via a non-cleavable maleimidocaproyl linker (38). MMAF preferentially acts on proliferating cells due to its antimitotic mechanism of action. This ADC was very potent in several tumor models inducing long-term regressions with low doses. In a NSCLC xenograft model the ADC reduced CSC frequency. In safety studies in primates it was safe and had a half-life of 5 days.

In the following, PF-06263507 was tested in combination with the dual phosphoinositide 3-kinase (PI3K)/mechanistic target of rapamycin (mTOR) catalytic site inhibitor PF-384 or taxanes (65). *In vitro*, PF-06263507 or untargeted auristatins displayed strong synergistic or additive activity when combined with PF-384 or taxanes, respectively. These synergistic/additive activities were not due to the individual components of the combination acting on different tumor cell subpopulations (e.g., CSC and non-CSC cells) but, rather, to amplification of the effects of this ADC on translational components by PF-384 or the simultaneous binding of MMAF and taxanes to distinct binding sites on microtubules, respectively. In human breast and lung cancer xenografts, combination therapy with PF-06263507 + PF-384 or PF-06263507 + paclitaxel yielded enhanced antitumor effects with longer survival as compared with monotherapies.

The potential of PF-06263507 for the treatment of hematological malignancies was also investigated (66), given the finding that 5T4 was overexpressed on minimal residual acute lymphoblastic leukemia (ALL) cells. PF-06263507 significantly improved survival in mice engrafted with 5T4^+^ patient-derived ALL cells, and even more so in combination with chemotherapy or dexamethasone.

PF-06263507 has been investigated in a phase I clinical trial (NCT01891669) and the results of this study, where no objective responses were observed, have been reported (39).

In another approach, anti-5T4 ADCs were constructed by site-specifically conjugating a human anti-5T4 mAb via a valine-alanine dipeptide linker with payloads having different mechanisms of action: a PBD dimer or the microtubule destabilizing tubulysin (40). *In vivo* experiments in xenograft models of different carcinoma types showed that the ADC conjugated with a PBD payload, MEDI-0641, elicited more durable antitumor responses and inhibited more potently the growth of 5T4^+^ CSCs *in vivo* than the tubulysin conjugate. This result is consistent with the knowledge that CSCs comprise subpopulations of proliferating and quiescent cells (67) and that a DNA binder like a PBD is cytotoxic also on quiescent cells, while a tubulin binder like tubulysin acts only on the proliferating CSC subpopulation. Moreover, MEDI-0641 was cytotoxic on both CSC and non-CSC tumor cells, leading to depletion of both compartments. This result implies that 5T4 is pan-tumor cell marker, whether or not overexpressed by CSCs. In rats, MEDI-0641 had excellent *in vivo* stability and an acceptable safety profile.

MEDI-0641 was then tested on cells and xenografts of head and neck squamous cell carcinoma (HNSCC) (41). *In vitro*, it caused a significant depletion of CSCs. *In vivo*, in three patient-derived xenograft models of HNSCC, a single administration of MEDI-0641 caused long-lasting tumor regression, which was likely due to depletion of both CSCs and non-CSCs. In the three models, MEDI-0641 caused either a complete elimination of tumor-initiating cells or a significant reduction. Moreover, a single dose of MEDI-0641 prevented tumor recurrence when used in a neoadjuvant setting prior to surgery.

MEDI-0641 does not seem to have yet entered clinical development as it is not mentioned neither under clinicaltrials.gov nor in the company's website.

A third anti-5T4 ADC has been described very recently (42). This ADC, H6-DM4, is composed of a chimeric anti-5T4 mAb linked to an antimitotic cytotoxin, the maytansinoid DM4, through a cleavable N-succinimidyl 4-(2-pyridylothio)butyrate (SPDB) linker sensitive to intracellular reducing conditions with liberation of a lipophilic adduct, S-methyl-DM4, that can exert a bystander effect. H6-DM4 was cytotoxic against a panel of gastrointestinal cancer cell lines, including colorectal CSCs and colorectal cancer cells resistant to platinum compounds. CSCs were found to express higher levels of 5T4 than non-CSCs. 5T4 eradicated established gastrointestinal tumor xenografts in the low mg/kg range without observable toxicity. Tumor cell lines expressing higher levels of 5T4 were more sensitive to the effects of the conjugate *in vivo*, suggesting that the expression level of 5T4 could represent a predictive marker for patient selection.

#### Anti-leucine-rich Repeat-Containing, G Protein-Coupled Receptor 5 (LGR5) ADCs

LGR5 is a marker of adult stem cells in several epithelial tissues. In particular, LGR5^+^ crypt cells in the gastrointestinal tract give rise to all differentiated cell types within intestinal epithelia, suggesting that it represents the stem cell of the small intestine and colon (68). LGR5 is overexpressed and gives rise to multiple cell types within gastrointestinal tumors (68, 69). These observations suggest a close relationship between normal adult gastrointestinal stem cells and CSCs of gastrointestinal tumors. LGR5 overexpression correlates with higher incidence of metastasis, drug resistance, and poor patient survival (70, 71). Given its overexpression and rapid, constitutive internalization independently of ligand binding (72), LGR5 was considered a good target for ADCs.

Two ADCs were constructed by conjugating an anti-LGR5 mAb to the membrane-permeable, antimitotic agent MMAE via a cleavable valine-citrulline linker or a non-cleavable malemidopropionyl linker (43). Both ADCs bound LGR5 with similar affinity and were rapidly internalized by gastrointestinal cancer cells. Cytotoxicity was induced in LGR5-overexpressing cancer cells, but not in LGR5-negative cells or cell lines where *LGR5* had been knocked down. However, the ADC with the cleavable linker was 10- to 20-fold more potent at killing these cells, probably due to a bystander effect (73). In fact, the ADC with the non-cleavable linker gives rise to charged metabolites (e.g., amino acid-linker-cytotoxin) that are membrane-impermeable. The ADC with the cleavable linker eradicated tumors and prevented recurrence in a xenograft model of colon cancer. Interestingly, these experiments yielded evidence of an interconversion between LGR5^+^ and LGR5^−^ CSCs that drove tumor regrowth after treatment with the ADC.

Another group has described the generation and testing of two other anti-LGR5 ADCs (44). The first is composed of a humanized anti-LGR5 mAb conjugated to MMAE through a cleavable valine-citrulline linker, via the cysteines that normally form the interchain disulfides of the mAb. Thus, this conjugate is very similar to the one described by Gong et al. (43). The second conjugate, NMS818, is composed of the same mAb connected, via an engineered cysteine on the antibody heavy chain and an acid-sensitive linker, to the C-14 hydroxyl of the DNA-binding, topoisomerase-inhibiting anthracycline PNU159682.

*In vivo* experiments showed the ADC anti-LGR5-MMAE to be efficacious without affecting homeostatic epithelia or any other tissues known to express LGR5. On the other hand, the ADC NMS818 showed target-dependent toxicities consistent with the known expression patterns of LGR5. The lack of gut toxicity with anti-LGR5-MMAE may possibly be due to the fact that the elimination of intestinal LGR5^+^ cells is well-tolerated. On the other hand, NMS818 target-dependent toxicity observed in the intestine may be attributable to the combined elimination of target-expressing LGR5 cells and bystander cells. In fact, both ADCs release a membrane-permeable drug after internalization that could exert a bystander effect on neighboring cells. However, the free drug released from NMS818 is 10- to 100-fold more potent on dividing cells, including normal LGR5^+^ cells, than MMAE. This could explain the greater toxicity of anti-NMS818 than anti-LGR5-MMAE.

Antitumor efficacy was observed both in xenografts as well as in genetically engineered mouse models of colon cancer, and both in tumors with uniformly high expression of LGR5 as well in tumors with heterogeneous and low expression of LGR5, the latter reflecting more closely the situation found in human tumors. Importantly, changes in tumor size were not immediately apparent, but became evident with long-term treatment, suggesting that depletion of CSCs takes longer to manifest as compared to the targeting of non-CSCs.

While there are clinical trials ongoing with a monospecific and a bispecific anti-LGR5 mAb, no trials are currently reported with one of the ADCs described here.

### Antitumor ADCs That Were Shown to Have Anti-CSC Activity at a Later Stage

#### Anti-human Epidermal Growth Factor Receptor 2 (HER2) ADC

HER2 is a transmembrane receptor tyrosine kinase that mediates several functions like growth, differentiation and survival in malignant and normal breast epithelial cells. Breast cancers overexpressing HER2 have an aggressive clinical phenotype, increased disease recurrence and unfavorable prognosis. Moreover, HER2 is overexpressed on breast CSCs, even on those subtypes that are not classified as HER2^+^ (74).

HER2 is the target of two antibody-based compounds that have gained approval: the first if the mAb trastuzumab (75), the second is the ADC ado-trastuzumab emtansine, which is composed of trastuzumab conjugated through a non-cleavable linker to the antimitotic drug maytansine DM1 (27). Clinical use of these compounds is for HER2^+^ breast cancer, with HER2 overexpression on the bulk of tumor cells, independently of their co-expression on CSCs.

The knowledge that CSCs overexpress HER2, whether or not the bulk of tumor cells are HER2^+^, led to investigate the effect of T-DM1 on CSCs (45). For this purpose, primary tumor cells and breast cancer cell lines were treated with T-DM1. The results showed that breast CSCs with the CD44^high^CD24^low^HER2^low^ phenotype were very efficient in internalizing T-DM1 and highly sensitive to it. This caused the depletion of breast CSCs at concentrations of T-DM1 that did not affect the bulk of tumor cells. Moreover, colony formation was also efficiently suppressed and EMT-mediated induction of stem cell-like properties was prevented in differentiated tumor cells. Importantly, the unconjugated antibody, trastuzumab, did not have these effects, pointing to a direct effect of the payload-induced cytotoxicity in depleting CSCs.

#### Anti-CD33 ADC

Gemtuzumab ozogamicin (GO) is an anti-CD33 ADC composed of a humanized anti-CD33 mAb linked to the cytotoxin calicheamicin via a hydrazone linker. GO has a tormented history. It received US Food and Drug Administration approval in 2000 for CD33^+^ AML, but was voluntarily withdrawn in 2010 due to safety concerns. Later, in a meta-analysis of patient data from clinical trials, it was found that the combination of lower-dose GO and induction chemotherapy reduced the risk of relapse and improved the relapse-free survival and overall survival in adult AML patients with favorable cytogenetics (76). These results led, recently, to the reapproval of GO.

Induction chemotherapy based on daunorubicin and cytarabine was investigated in combination with GO in patient-derived xenograft AML models (46). The separate treatments reduced AML burden but left significant chemoresidual disease. Chemoresistant cells displayed markers of LSCs and showed greater ability to self-renewal than bulk leukemic cells. Interestingly, CD33 was coexpressed in the chemoresistant cells. Combination treatment, on the other hand, was highly effective in eliminating nearly all AML burden, extended overall survival and more effectively eliminated chemoresistant LSCs.

#### Anti-neural Cell Adhesion Molecule (NCAM, CD56) ADC

Wilms' tumor is the most frequent tumor of the genitourinary tract in children. It displays a triphasic histology: cell lineages similar to those observed during kidney development, undifferentiated blastema and stromal and epithelial derivatives (e.g., immature tubules and glomeruloid bodies). Evidence for the existence of CSCs in human Wilms' tumor was obtained in *in vitro* cultures derived from primary tumors. In these experiments it was found that NCAM^+^ cells of blastema phenotype had enhanced capacity to expand and differentiate into mature renal-like cell types, showing that they were greatly enriched for CSCs (77). They could be further enriched by aldehyde dehydrogenase activity and overexpression of other stemness genes and showed preferential expression of Akt and strong reduction of the miR-200 family, a miRNA family involved in the down-regulation of EMT and maintenance of an epithelial phenotype (78).

In order deplete NCAM^+^ cells in Wilms' tumors an ADC was used that had already been constructed and developed, lovortuzumab mertansine (47). This ADC is composed of a humanized anti-NCAM mAb, lovortuzumab, linked via a cleavable disulfide linker to the maytansinoid DM1, mertansine. *In vitro*, it inhibited the stemness properties of Wilms' tumor cell cultures that varied in the extent of NCAM expression. Results suggested also that EMT promoted the acquisition of a CSC phenotype generating highly tumorigenic cancer cells with a mesenchymal phenotype. *In vivo*, the ADC eradicated, at low doses, Wilms' tumors bearing high NCAM expression, while higher doses were required for Wilms' tumors with lower NCAM expression.

The clinical development of this ADC has recently been halted due to disappointing results in a clinical study in lung cancer patients (48).

## A Critical Appraisal of Anti-CSC ADCs

ADCs against CSCs, similarly to other anti-CSC compounds, have raised considerable hopes as regards their therapeutic efficacy. Recently, however, one of the most advanced ADCs of this class, the anti-DLL3 ADC rovalpituzumab tesirine, has yielded disappointing results in a clinical trial (NCT02674568) in patients with SCLC (30). This is despite encouraging results in an initial phase I clinical trial in the same indication (79). This failure parallels other pitfalls with drug candidates targeting CSCs, including other ADCs. The question now arises whether these approaches, including the ADC approach against CSCs, have entered a dead-end street or if there is room for envisioning a new start based on a better understanding of the reasons underlying these failures. In the following we will list some of the reasons that appear to us as the most likely ones.

The first aspect relates to the biology of CSCs. CSCs are themselves a heterogeneous population that can be grossly divided in two subpopulations: a proliferating and a quiescent subpopulation. There are strong indications that the quiescent subpopulation is in an autophagic state (67). Importantly, there are also evidences that these two subpopulations may occupy different niches within tumor tissues (80). The location of a tumor cell within a tumor is crucially important for their sensitivity to drugs, including antibodies and ADCs (81). This implies that, depending on their location, different CSC subpopulations may be differently sensitive to the same drug.

The existence of proliferating and quiescent CSC subpopulations implies also that ADCs carrying cell cycle-independent drugs like DNA binders have an advantage over cell cycle-dependent drugs like tubulin binders and may lead to a more complete elimination of CSCs because of their potential to delete both proliferating as well as quiescent cells. If, however, quiescent CSCs are in an autophagic state, and there is considerable evidence in favor of this possibility (reviewed in 40), then there is another dark side that we should consider because our knowledge on this aspect and the possible consequences is almost nil. In fact, we don't know whether the internalization and intracellular trafficking in autophagic cells follows the same kinetics and routes as that of the non-autophagic counterparts. Recent evidence suggests, indeed, that there are differences (82). This implies that these differences may cause in autophagic CSCs to a release of the active drug that is less efficient that in proliferating CSCs.

Another limitation of the ADCs that are currently developed is that none of them targets a marker that is strictly CSC-specific. As already discussed before, these targets are also expressed, albeit to a lesser degree, on the bulk of tumor cells. This is notwithstanding the lack of reactivity of these ADCs for normal stem cells and the lack or limited reactivity for normal tissues. At this point one is led to ask whether these ADCs are truly anti-CSC ADCs or anti-tumor cell ADCs that embrace a tumor cell population that includes also CSCs. This is not necessarily a disadvantage, but then one has to evaluate the overall antitumor activity of the ADC in order to predict its efficacy and not just the anti-CSC activity.

One of the most important, yet disregarded aspects of CSC biology lies in the capacity of CSCs and non-CSCs to interconvert. This aspect has already been briefly addressed in the first section of this article. Nevertheless, it is important, at this point, to underscore the consequences that this may have on the whole tumor cell population and on CSCs. We have discussed that the conversion from non-CSC to CSCs can be driven by stressors in the tumor microenvironment, including hypoxia, mechanical stress, chemotherapy etc. These stressors may show inter- and even intratumoral variability depending on the geographical conditions of the tumor microenvironment. This implies that the fraction of CSCs may greatly vary from one tumor to the other and even within individual tumors. Such great variability has been documented in several instances (83, 84) and, in some cases, one out of four tumor cells have been shown to display properties of CSCs (85). It is clear that in these cases the boundaries between non-CSCs and CSCs become very blurred. The capacity of CSCs and non-CSCs to interconvert represents a substantial difference compared to normal stem cells. In fact, normal stem cells reside at the top of a pyramid where they can self-renew or give rise to a more differentiated progeny (86). This implies a unidirectional process, whereas the capacity of CSCs and non-CSCs to interconvert implies a bidirectional process. This raises serious doubts as to the appropriateness to refer to these cells as stem cells. Perhaps a definition like “resistant cancer cells” would be more appropriate to portray the essential of these cells.

Last but not least, an important limitation in the development of antibodies and ADCs is represented by the animal models that are used for preclinical testing. As can be seen just going through the articles that are referenced here, it is rather quite common that the compounds tested in animal models lead to complete tumor regressions, yet the same compounds fail in the human setting (e.g., 31, i.e., the ADC rovalpituzumab tesirine). In other terms, the predictability of animal models is very limited and it seems appropriate to say that observing complete tumor regressions in animal models is, nowadays, a necessary but by far not sufficient condition. At this point, the obvious question arises as to why animal (essentially mouse) models are inadequate to predict efficacy of antibodies and ADCs? A detailed discussion of this aspect goes beyond the scope of this article. Nevertheless, we would like to briefly address two aspects that may contribute to this insufficiency. First, the percentage of injected dose of an antibody or antibody-like construct that is taken up by the tumor mass (expressed in grams) is much lower for humans than for mice (87). This is because an antibody dose diluted in a larger plasma volume gives a much lower percentage of injected dose/gram tumor tissue, three orders of magnitude lower for humans (~3 l plasma volume) than mice (~2 ml). Such a dose may be sufficient to eradicate a tumor in a mouse, but insufficient to eradicate an equivalent tumor in a human. Incidentally, it has been recently demonstrated that the intratumoral payload concentration correlates with the antitumor activity of ADCs (88), a result that underscores the importance of attaining a sufficient antibody or ADC concentration within a tumor tissue in order to be efficacious. The second aspect relates to the mouse tumor models that are commonly used to test antitumor drugs, including those that are considered mimicking most closely the human situation, PDXs. It seems very difficult that these models can fully mimic the heterogeneity encountered in the human setting, in particular as regards the possibility that stressors from the tumor microenvironment may induce a conversion of non-CSC to CSCs. Thus, in one of the articles that have been referenced here (41), the anti-5T4 ADC MEDI-0641 ablated CSCs in one PDX model of HNSCC, reduced the frequency of CSCs 5-fold in a second PDX model, and 2-fold in a third PDX model (M11). Yet, in spite of the relatively modest CSC reduction that was observed in the M11 model, MEDI-0641 was able to completely prevent tumor recurrence after surgical removal of the tumor in this model. This leads obviously to ask as to which is the predictability of ADC-induced reduction of CSC frequency on the *in vivo* efficacy of an ADC in PDX models.

In spite of all these limitations and even if we resort to a minimalist definition of CSCs as tumor cells that become drug-resistant in response to stressors generated in the tumor microenvironment, then we have to agree that it remains a desirable therapeutic goal to get rid of these cells together with the rest of the tumor cells, i.e., the proliferating, drug-sensitive tumor cells. The point is to how best attack these cells. Addressing this question, however, implies recapitulating all of the limitations that we have listed and discussed in the first part of this section. Now, how can we address this conundrum? There is no easy answer to this but, at present, we can envisage two possibilities that can to be implemented.

The first of these possibilities is to develop *in silico* models of human tumors that are better able to predict efficacy of antibodies or ADCs than do the present animal models. Efforts in this direction are ongoing (89). Thus, *in silico* models for tumor growth and tumor treatment have been described (90, 91). In perspective, it seems reasonable to predict that these models may incorporate a number of variables allowing them to match the situation(s) encountered in the human setting more closely than currently used animal models, including PDX models, are able to do.

Second, the identification of biomarkers that are predictive of clinical efficacy of anti-CSC ADCs, similarly to the overexpression of HER2 that is used to predict efficacy of trastuzumab in HER2^+^ breast cancer patients. Biomarkers, including circulating tumor cells (CTC), witnessing antitumor effects in general and anti-CSC effects in particular (e.g., CTCs with a mesenchymal phenotype or soluble markers from mesenchymal CSCs), may allow to predict in a more reliable manner the overall clinical efficacy of anti-CSC ADCs. Such an approach could be systemically applied to the anti-CSC ADCs that are currently in clinical development. Elucidation of the relationship/lack of relationship between anti-CSC activity and clinical efficacy could allow to identify the contribution of anti-CSC activity to the overall antitumor activity and predict the efficacy of novel ADCs.

Overall, the present scenario regarding the clinical efficacy of anti-CSC ADCs is not very encouraging, yet the biological functions of CSCs suggest that further efforts should be devoted to this goal. Here, we have proposed some activities that could help moving forward in this field.

## Author Contributions

FM, CC, ER, and CR contributed to the conception of the work, drafted or revisited it critically, approved the final version, and agreed to be accountable for all aspects of the work.

### Conflict of Interest Statement

The authors declare that the research was conducted in the absence of any commercial or financial relationships that could be construed as a potential conflict of interest.

## Abbreviations

ADC: antibody-drug conjugate
ALL: acute lymphoblastic leukemia
AML: acute myeloid leukemia
CSC: cancer-stem like cell
DAR: drug-antibody ratio
DLL: Delta-like ligand
EFNA4: Eph-A4
EMT: epithelial-mesenchymal transition
Eph: Ephrin receptor
FLT3: fms-like tyrosine kinase 3
GO: gemtuzumab ozogamicin
HER2: human epidermal growth factor receptor 2
IL: interleukin
LRG5: leucine-rich repeat-containing; G protein-coupled receptor 5
LSC: leukemia stem cell
mAb: monoclonal antibody
MMA: monomethyl auristatin
mTOR: mechanistic target of rapamycin
NCAM: neural cell adhesion molecule
NSCLC: non-small cell lung cancer
PBD: pyrrolobenzodiazepine
PDX: patient-derived xenograft
PI3K: phosphoinositide 3-kinase
PTK: protein tyrosine kinase
SCLC: small cell lung cancer
SOC: standard-of-care
SPDB: N-succinimidyl 4-(2-pyridylothio)butyrate
TNBC: triple-negative breast cancer.
